# Combining Organic and Foliar Fertilization to Enhance Soil Fertility and Mitigate Physiological Disorders of Durian (*Durio zibethinus* Murr.) Fruit in the Tropics

**DOI:** 10.3390/plants14081185

**Published:** 2025-04-11

**Authors:** Le Van Dang, Nguyen Kim Quyen, Ngo Phuong Ngoc, Le Minh Ly, Pham Thi Phuong Thao, Ngo Ngoc Hung

**Affiliations:** 1 College of Agriculture, Can Tho University, Can Tho City 94000, Vietnam; lvdang@ctu.edu.vn (L.V.D.); npngoc@ctu.edu.vn (N.P.N.); minhly@ctu.edu.vn (L.M.L.); ptpthao@ctu.edu.vn (P.T.P.T.); ngochung@ctu.edu.vn (N.N.H.); 2 United Graduate School of Agricultural Science, Tokyo University of Agriculture and Technology, Tokyo 183-8509, Japan; 3 Faculty of Agriculture and Fishery, University of Cuu Long, Vinh Long 85000, Vietnam

**Keywords:** physiological disorders, plant nutrition, soil amendments, durian

## Abstract

Physiological disorders (PDs) in durian lead to reduced commodity prices; therefore, reducing the PD rate in durian enhances the fruit’s value and farmers’ profits. Nutrient and soil management may affect the PD rate during fruit development. Herein, we used amendments such as organic manure (OM) and foliar fertilization (FF) applications to reduce the PD rate and improve the soil health and fruit yield of durian. This study was conducted in three durian orchards in the Vietnamese Mekong Delta from 2022 to 2024. The treatments were as follows: (i) control (unamended), (ii) OM, (iii) FF, and (iv) OM + FF. N−P−K fertilizers (0.45 kg of N, 0.45 kg of P, and 0.45 kg of K per tree) were uniformly applied to all durian trees. We measured the characteristics of the soil, such as the soil pH, soil organic carbon (SOC), available phosphorus (AP), and exchangeable cations (K^+^ and Ca^2+^). The leaf nutrient (K and Ca) content, fruit yield (kg tree^−1^), and fruit quality (PD rate, total soluble solids (TSS), and aril color characteristics) were also recorded. Our study indicates that OM + FF increased soil pH and SOC, AP, and exchangeable cations (K^+^ and Ca^2+^). In addition, the K and Ca concentrations in durian leaves increased by approximately 4% using OM + FF. Combining OM and FF decreased the PD rate of durian fruit (>85%) compared with the control. This practice increased the fruit quality TSS (13%), color, proportion of arils, and fruit yield (~10%) compared with conventional practice (control). Overall, using OM and FF contributed to improving durian production and values. Therefore, we recommend that farmers who cultivate durian apply OM + FF to their orchards to enhance soil health, fruit quality, and yield.

## 1. Introduction

Recently, land-use conversion from rice fields to fruit orchards has been a standard approach in the Mekong Delta, Vietnam (VMD), because of the low income and benefits obtained from rice production compared with fruit crop production [1]. Durian (*Durio zibethinus* Murr.) is among the fruits with high economic value; it contributes to poverty reduction and enhances livelihoods in rural areas [2]. Therefore, the area of durian cultivation in the VMD has increased dramatically in the last 10 years, from 12,600 ha in 2013 to 33,200 ha in 2022 [3]. This area accounts for approximately 35% of the total durian production in Vietnam [3]. Durian offers a substantial income for growers, but various techniques are required to achieve high yields and economic efficiency [4]. As durian is a new fruit crop, farmers cultivating durian in the VMD region lack knowledge about its production, especially regarding nutrient and pest management, which leads to decreased fruit yield and quality.

According to Hieu and Hau [5], physiological disorders (PDs) in durian fruit are key factors in durian production in the VMD. They indicated that the high rates of PDs found in durian fruit are due to a lack of nutrients and high soil moisture before fruit harvesting. PDs decrease the color, flavor, and appeal of the arils, resulting in a reduction in the value and commodity prices for durian fruit. A recent study reported approximately 50% deficiencies in K and Ca in 180 durian leaf samples from this area [6]. These elements play an essential role in fruit quality, especially in alleviating the PD rate [7]. Hau et al. [8] observed that a decrease in soil moisture a month before fruit harvesting causes a reduction in the PD rate. Thus, well-controlled soil moisture and nutrient management could improve durian fruit quality. Our previous study indicated that plastic mulching is the best way to control soil moisture during heavy rains and improve the PD rate of durian fruit [7]. It also showed that applying polyhalite fertilizer containing K and Ca elements increased the contents of these elements in durian leaves, leading to a decreased PD rate and improved fruit quality [7]. However, the study focused on polyhalite fertilizer and did not evaluate the role of other fertilizers, such as organic manure or foliar fertilization, that may improve the PD rate by supplying various available elements (e.g., K and Ca).

Many studies have reported two pathways to crop nutrient supply: soil and foliar applications [9,10]. The first pathway, fertilization, refers to improving soil nutrient concentrations [11]. This method is primarily used in the agricultural system and contributes to plant growth and development because plants uptake and use nutrients through their roots [12]. The second pathway is supplementing nutrients via foliage, which allows the supplied elements to be absorbed directly through the stomata or leaf surface [13]. This method is usually used when plants face abiotic or biotic stresses, such as drought, salinity, or pest infestation [14]. Especially in the reproductive stage, plants need more nutrients to feed the fruit, and the nutrients supplied by the soil might not be enough to meet plant demand.

Organic manure (OM) application refers to soil feeding; it supplies nutrients and improves soil quality due to its high amounts of organic matter and available nutrients, including macro- and micro-nutrients [15,16]. OM is crucial in soil microbial activity, which significantly affects soil health [17,18]. In addition, organic materials enhance the soil structure, which helps roots to grow and develop well [19]. Moreover, OM can regulate and maintain the soil pH, affecting crops’ available nutrients [20]. The VMD is mainly located in tropical climate areas, which are notable for their high precipitation and low soil pH. However, information on the role of OM in improving soil acidity, soil fertility, and durian fruit yield and quality is limited.

Likewise, foliar fertilization (FF) is essential in fruit production, especially in the fruit development stage [21,22]. Previous studies have shown that foliar application can quickly address nutrient deficiencies compared to soil feeding [14,23]. This method can be effectively used to supply macro- and micro-elements in poor root growth conditions. Additionally, it can be sprayed together with other soluble agrochemicals [24]. This combined approach can significantly decrease the cost of production, thus increasing the farmer’s revenue. Furthermore, it reduces environmental issues, such as nutrient losses and volatilization [14]. A previous study indicated that using FF in citrus orchards improved the leaf nutrient content and fruit quality [25]. According to Ishfaq et al. [14], foliar feeding increased the crop quality and yield by approximately 20%. However, the effects of FF on the PD rate and fruit quality of durian remain unclear.

Therefore, our study aimed to evaluate the influence of OM and FF applications on soil quality, the durian fruit PD rate, and yield cultivation in the VMD. We hypothesized that (i) using OM could increase the soil pH and soil nutrient contents; (ii) FF supplementation might improve leaf nutrient contents, which could decrease the PD rate; and (iii) combined OM and FF could increase soil fertility and leaf nutrient contents, leading to an increase in durian fruit quality and yield.

## 2. Results

### 2.1. Changes in Soil Quality Characteristics (0–20 cm) and Leaf Nutrient (K and Ca) Concentrations Under Different Amendments

#### 2.1.1. Soil Characteristics

Using OM combined with FF significantly improved the soil’s chemical quality at all study sites in both seasons (Table 1). Applying OM or OM + FF increased the soil pH by approximately 0.2 units compared with the control and FF treatments. Similarly, the SOC concentration was significantly enhanced (3~4 g C kg^−1^) when OM or OM + FF was used. Applying OM or OM + FF increased the AP (by 3~5 mg P kg^−1^) compared to FF and the control. Likewise, soil exchangeable cations (K^+^ and Ca^2+^) were positively affected by OM and OM + FF. In particular, the soil exchangeable K^+^ content increased by around 0.1 cmol_c_ 100 g^−1^ after OM or OM + FF addition. These same practices increased the soil exchangeable Ca^2+^ content by approximately 0.5 cmol_c_ 100 g^−1^. In short, combining OM and FF improved soil quality properties compared to the unamended samples (control).

#### 2.1.2. Leaf Nutrients

Table 2 shows the concentrations of K and Ca in durian leaves at three study locations over two seasons (2022–2023 and 2023–2024). Using OM combined with FF significantly improved the contents of both nutrients (K and Ca) in durian leaves, increasing them by approximately 4 g K kg^−1^ and 5 g Ca kg^−1^, respectively. Adding OM or FF individually also increased the concentrations of leaf K and Ca compared with the control. However, combining OM and FF enhanced the durian leaf K and Ca contents more efficiently.

### 2.2. Fruit Yield and Quality Under Organic Manure and Foliar Fertilization

#### 2.2.1. Fruit Proportions

Combining OM and FF significantly decreased the durian peel proportion compared to the control, OM, and FF treatments (Table 3). The peel proportion was reduced by approximately 4% after the use of OM + FF at the three study sites in two consecutive seasons. Meanwhile, we observed no significant difference in the durian peel proportion among the OM, FF, and control treatments. The results showed that the durian seed proportion was not affected by the amendments, accounting for around 8% of the total durian fruit in each case. We found that the aril proportion increased significantly in the OM, FF, and OM + FF treatments compared with the control. However, the combination of OM and FF led to the highest aril proportion: around 28% of the total durian fruit. In conclusion, using OM and FF decreased the fruit peel proportion but increased the fruit aril proportion.

#### 2.2.2. Fruit Yield and Quality

Table 4 shows the mean values for durian fruit yield and quality results under the different amendments. Combining OM and FF increased the fruit yield by approximately 10% compared with the control in both seasons in the three durian orchards. Using OM or FF alone also improved the fruit yield but to a lesser extent than OM + FF. Similarly, the combination of OM and FF significantly increased the b* value of the arils compared with the other treatments (control, OM, and FF). Likewise, the TSS concentration was enhanced by around 13% under the OM + FF treatment. In contrast, the application of OM and FF reduced the PD rate by ~85% compared with the control. In short, combining OM and FF increased the fruit yield, b* value, and TSS content and decreased the PD rate.

### 2.3. Relationships Between Soil and Plant Characteristics

We observed that there were positive correlations between soil pH and AP (r = 0.87 **) and between soil pH and soil exchangeable Ca^2+^ (r = 0.77 **) (Table 5). Similarly, SOC was positively related to AP, soil exchangeable Ca^2+^, and K^+^. In contrast, there were negative correlations between the PD rate and soil pH, SOC, AP, soil exchangeable Ca^2+^ and K^+^, and leaf K and Ca, with r values of −0.54, −0.71 **, −0.52 *, −0.54 *, −0.46 *, −0.88 **, and −0.91 **, respectively. In short, most soil and plant parameters were negatively related to the PD rate.

## 3. Discussion

The PD rates decreased significantly after the application of OM and FF because combining OM and FF enhanced soil quality (Table 1) and optimized leaf nutrients (Table 2). In addition, fruit yield and proportions also improved significantly when using OM and FF (Table 3 and Table 4). Although the use of OM alone also improved soil quality, FF increased leaf nutrient content; however, its efficiency in reducing PD rates was lower than that of combining OM and FF. These results demonstrate the synergy of OM and FF in mitigating PD rates in durian fruit.

In this study, we found that enhancing the soil pH increased the availability of P, improving the fruit quality by reducing the PD rate (Table 5). According to Wu et al. [26], P plays an essential role in fruit growth and development. In addition, it regulates fruit quality through its primary role in energy transformation and photosynthesis transportation [27,28]. Zhang et al. [28] reported that P plays a crucial role in synthesizing enzymes that take part in starch formation. Additionally, P is essential for the production of amino acids and properly functioning ribosomes during protein synthesis [29]. Reducing the starch and protein contents in the arils could increase the PD rate due to decreased aril quality (dryness and hardness). Indeed, we found a low aril proportion in the control treatment (Table 3). Unfortunately, we did not measure the starch and protein contents in the arils. However, a previous study indicated that low starch and protein contents increased the unevenness of fruit ripening in durian [7]. In short, improving the availability of soil P decreased the PD rate.

This study indicates that combining OM and FF significantly increased the SOC concentration during two consecutive seasons (Table 1). SOC is essential in soil health and root growth by supplying available nutrients and improving soil porosity [30,31]. Indeed, a positive correlation was found between SOC and soil exchangeable cations (K^+^ and Ca^2+^) (Table 5). According to Duong et al. [32], soil nutrients are released during the decomposition of organic matter. They are in a soluble form and are available for plants; therefore, they are more easily taken up, resulting in improved yield and quality. In addition, in this study, we observed that enhancing the SOC contributed to increasing the content of available P (positive correlation, r = 0.77 **). As mentioned above, available P is essential in decreasing the PD rate. Therefore, improving SOC contributes to reducing the PD rate. In addition, the OM used in this study contained high amounts of exchangeable K^+^ and Ca^2+^ cations (15.0 and 61.6 cmol_c_ kg^−1^, respectively). This factor contributed to the increase in these elements in the soil (Table 1). The results are in line with those from Nurdianto et al. [33], who found that adding organic fertilizer containing high amounts of base cations such as K and Ca significantly increased the contents of these nutrients in the soil after 5 months of application. Various studies have found that OM + FF increases crop quality [15,34]. According to Ye et al. [35], the content of TSS in pears increased significantly after the application of OM and FF, resulting in increased fruit quality. Our previous studies [25,36] showed that the quality of citrus fruits significantly increased after the application of OM + FF due to an improvement in the TSS content and a decrease in titrate acidity. The current study indicates that using OM + FF increases the TSS concentration in durian arils (Table 4). Therefore, this practice should be applied to enhance durian fruit quality cultivation in the VMD.

The leaf K and Ca concentrations significantly increased under the OM + FF treatment (Table 2). This was due to the durian plants receiving more K and Ca from OM and FF together than from other treatments. Indeed, applying OM or FF alone also improved the leaf K and Ca contents compared with the control but to a lesser extent than their combination. This study shows a strong negative correlation between leaf nutrient contents (K and Ca) and the PD rate (Table 5). According to Hau et al. [8], enhancing durian leaf K and Ca contents significantly decreases the PD rate. K and Ca regulate biochemical processes in plants, contributing to the production of carbohydrates (starch and sugar) [37,38]; therefore, they directly affect the flavor and color of durian arils. Our previous study indicated that improving the concentrations of K and Ca in durian leaves significantly decreases the uneven fruit ripening rate [7].

Overall, we observed negative correlations between soil characteristics, leaf nutrients, and the PD rate (Table 5). In other words, increasing the soil and leaf quality reduced the PD rate in durian. These results indicate a synergetic effect of OM and FF on improving fruit yield and quality. Therefore, farmers should use these amendments in combination in their durian orchards to enhance fruit yield and decrease the PD rate. Although the application of both OM and FF demonstrates efficiency in durian production, more studies on its economic efficiency are necessary to evaluate farmers’ profits and production avenues.

## 4. Materials and Methods

### 4.1. Study Location, Soil, Plants, and Climate

**Figure 1:**
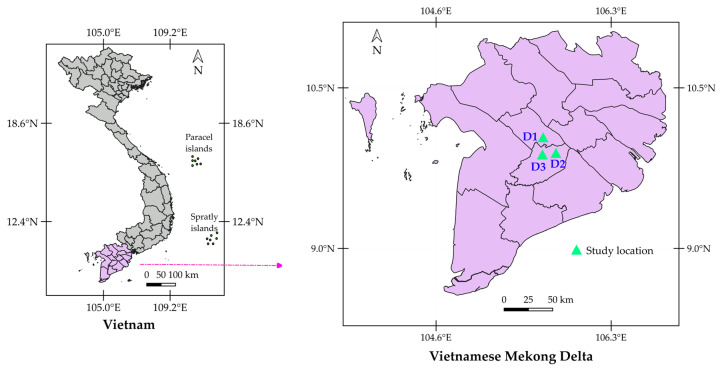
The locations of experimental sites.

The study sites were located at (1) D1 (Phong Dien District, Can Tho City, 10°01′12″ N 105°37′38″ E), (2) D2 (Chau Thanh District, Hau Giang Province, 9°57′15″ N 105°45′21″ E), and (3) D3 (Phung Hiep District, Hau Giang Province, 9°54′51″ N 105°43′31″ E). Figure 1 shows the locations of the three durian orchards.

The detailed initial soil properties (0–20 cm) for the three study locations are presented in Table S1. Briefly, the soils were classified as alluvial soils by referring to the FAO classification [39], with low pH (<5.0) and SOC content (<20 g C kg^−1^). Medium soil AP content (20.5–25.9 mg P kg^−1^) and soil bulk density (1.05–1.11 g cm^−3^) were observed. The soil exchangeable cation (Na^+^, K^+^, Ca^2+^, and Mg^2+^) contents ranged within 0.35–0.44, 0.52–0.66, 4.85–5.33, and 5.09–5.77 cmol_c_ 100 g^−1^, respectively. Sand, silt, and clay accounted for 0.8–1.3%, 44.3–45.5%, and 53.2–54.9% of the soil particles, respectively.

A “Ri 6” durian cultivar orchard was selected for each study site because this cultivar is popular in the VMD and accounts for 70% of the durian cultivation area [6]. Durian orchards (6 years old) with areas of 0.4–0.5 ha (110~140 durian trees, 6 × 6 spacing) were chosen for the experiment. The durian plants used in the experiment had similar trunk and stem diameters. Pest and weed control were performed uniformly for all durian trees. The average monthly rainfall during the experimental period was approximately 150 mm [7]. High rainfall was recorded from May to November, with a mean of around 240 mm per month [7].

### 4.2. Organic Manure and Foliar Fertilization

This study utilized a commercial organic fertilizer product from PPE Co., Ltd., Can Tho City, Vietnam, with sugarcane filter cake as the primary feedstock. The nutrient content in this manure was reported in a previous study [40]. In detail, the total C and porosity were 15.4% and 76.2%, respectively; the concentrations of exchangeable K^+^ and Ca^2+^ cations were 15.0 and 61.6 cmol_c_ kg^−1^, respectively; and the available P content was 3.6 g kg^−1^. Foliar fertilizer (TANO 708) was obtained from Dai Nghia Biotech Co., Ltd., Ho Chi Minh City, Vietnam. According to the producer, it contains 4% K_2_O and 4.3% Ca and is specialized for durian use.

### 4.3. Experimental Design

The study was carried out in three different durian orchards in two seasons (2022–2023 and 2023–2024). The experiment was based on a randomized complete block design in each orchard. The treatments were (i) the control (unamended), (ii) OM, (iii) FF, and (iv) OM + FF. Each treatment consisted of three replicates with 12 durian trees. The amount of OM was 5 Mg ha^−1^ season^−1^, and OM was applied 15 days after durian fruit set, using the topdressing method. Based on the producer’s recommendations, FF was supplied three times a season through spraying at 40, 60, and 80 days after fruit set.

N, P, and K fertilizers were additionally applied for all durian trees, with a dose of 0.45 N − 0.45 P − 0.45 K (kg tree^−1^), using N–P–K (15–15–15) complex fertilizer. They were separated into three stages throughout durian fruit development. The first stage was 25 days after fruit set, with 30% N–P–K fertilizer applied. The second stage was 50 days after fruit set, with 40% N–P–K fertilizer added. The final stage was 35 days before fruit harvest, with 30% N–P–K fertilizer supplied. As with the OM, we used the topdressing method to add the N–P–K fertilizer.

### 4.4. Soil and Durian Leaf Collection and Analysis

We initially planned to collect soil samples at two depths (0–20 and 20–40 cm). However, our previous study [7] indicated that nutrient application (the topdressing method) to the surface soil did not affect soil properties in the 20–40 cm soil layer. Therefore, we only collected soil samples at 0–20 cm depths during the fruit harvesting period. We used a soil auger (Φ = 17 cm) to collect samples around the durian canopy at three points, spaced 1 m from the durian trunk. They were mixed into one sample, from which approximately 300 g was taken and put into a plastic bag. A total of 72 soil samples (4 treatments × 3 replicates × 3 sites × 2 seasons) were collected in the current work. Afterwards, all samples were air-dried in a room at 25 °C for over 15 days; then, the soil samples were crushed and sieved through 0.5 and 2 mm meshes. We analyzed the soil parameters with reference to Houba et al. [41]. Soil pH was measured with a digital pH meter using a 1:2.5 soil-to-water ratio. SOC was determined using the Walkley–Black method, which involves extraction with 10 mL of K_2_Cr_2_O_7_ and 20 mL of 96% *w*/*w* H_2_SO_4_, followed by titration with 0.5 M FeSO_4_. Exchangeable cations (Ca^2+^ and K^+^) were extracted using 0.1 M BaCl_2_ and quantified with an Atomic Absorption Spectrometer (iCE 3500, Thermo Scientific, Waltham, MA, USA).

We collected the durian leaf samples simultaneously with the soil sampling. Leaves were collected at the 5th and 6th positions of the bud. Six leaves were picked from each durian tree, so twenty-four leaves were collected in each replication. We carefully washed the leaves with distilled water to eliminate dust and insects on the leaf surface. Then, the leaves were placed in an oven at 70 °C for six days and crushed using powder machinery. After that, the leaf K and Ca concentrations were determined following the guidance of Houba et al. [41]. Firstly, samples were converted into inorganic forms using a chemical compound containing salicylic acid (6 g), deionized water (18 mL), and H_2_SO_4_ 96% (100 mL). A plastic dropper pipette was used to add 15 mL of H_2_O_2_ (30%) throughout the digestion period. Finally, the leaf K and Ca contents were determined using an Atomic Absorption Spectrometer (iCE 3500, Thermo Scientific, Waltham, MA, USA) at 766.5 nm and 422.7 nm wavelengths, respectively.

### 4.5. Evaluation of Fruit Yield and Quality Characteristics

**Figure 2:**
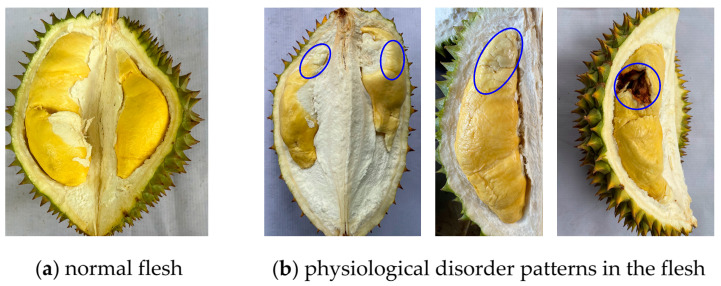
These photos illustrate normal flesh (**a**) and physiological disorders (**b**) in durian “Ri 6”. The blue circles indicate areas showing physiological disorder symptoms.

We weighed all the durian fruits (fresh weight) during the fruit harvesting period to record the fruit yield (kg tree^−1^). The data on fruit yield were evaluated in two seasons (2022–2023 and 2023–2024). Likewise, the fruit quality was measured in both seasons. Firstly, we randomly selected three durian fruits in each replicate to evaluate their quality. Each durian fruit ranged from 2.2 to 2.8 kg in weight and had no physical damage due to pest infestation. We visually evaluated the physiological disorder (PD) rate after opening the durian fruits (Figure 2). We recorded the total number of flesh components (arils + seeds) in each durian fruit and then determined the PD percentage based on the number of flesh components exhibiting a PD divided by the total number of durian flesh components. Subsequently, the durian fruits were separated into three parts: arils, seeds, and peel. The parts were weighed to establish their representative proportions in the total mass of the fruit. Next, the durian aril color and total soluble solid (TSS) content were assessed. The detailed methods for determining these characteristics were described in our previous study [7]. Briefly, a handheld colorimeter (CR-20 Color Reader, Konica Minolta, Tokyo, Japan) was used to measure the colors of the durian arils. The values of L* (lightness intensity), a* (+redness, −greenness), and b* (+yellowness, −blueness) were recorded. An instrument (Atago PAL−1 Pocket Refractometer, Saitama, Japan) was used to determine the TSS content in the durian arils [7].

### 4.6. Data Analysis

IBM SPSS statistical software (version 20) was used for data analysis. We used the Duncan post hoc test (*p* < 0.05) to determine significant differences among the soil amendment treatments in terms of soil and leaf parameters, as well as fruit quality and yield. Furthermore, we analyzed the relationships between all parameters using Pearson’s correlation test.

## 5. Conclusions

Our study indicated that applying organic manure (5 Mg ha^−1^ season^−1^) and foliar fertilization (TANO 708) improved soil quality, leaf nutrients, and durian fruit yield cultivation in the VMD. This practice contributed to a decrease in the rate of physiological disorders in durian fruits. These results suggest that durian growers should use a combination of organic manure and foliar fertilization to enhance durian fruit quality in their orchards. This could improve the value of durian in the VMD, increasing farmers’ profits.

## Figures and Tables

**Table 1:** Mean values for soil chemical characteristics under different amendments.

Site	Season	Treatment	Soil pH H_2_O (1:2.5)	SOC (g C kg^−1^)	AP (mg P kg^−1^)	Ex. K^+^ (cmol_c_ 100 g^−1^)	Ex. Ca^2+^ (cmol_c_ 100 g^−1^)
D1	2022–2023	Control	4.75 b ± 0.08	20.3 b ± 0.85	22.5 b ± 0.62	0.50 b ± 0.02	4.79 b ± 0.06
		OM	5.04 a ± 0.11	23.9 a ± 0.70	26.7 a ± 0.46	0.60 a ± 0.02	5.19 a ± 0.04
		FF	4.77 b ± 0.08	20.2 b ± 0.35	22.3 b ± 0.40	0.52 b ± 0.03	4.76 b ± 0.07
		OM + FF	5.06 a ± 0.06	24.3 a ± 0.42	26.3 a ± 0.56	0.62 a ± 0.03	5.26 a ± 0.06
		*p*-value	**	***	***	**	***
	2023–2024	Control	4.95 b ± 0.07	19.2 b ± 0.32	22.7 b ± 0.38	0.52 b ± 0.02	4.75 b ± 0.09
		OM	5.13 a ± 0.10	23.9 a ± 0.65	26.4 a ± 0.42	0.62 a ± 0.03	5.22 a ± 0.09
		FF	4.88 b ± 0.09	19.7 b ± 0.32	22.6 b ± 0.55	0.54 b ± 0.04	4.78 b ± 0.09
		OM + FF	5.17 a ± 0.03	24.8 a ± 0.74	26.0 a ± 0.47	0.63 a ± 0.04	5.25 a ± 0.06
		*p*-value	***	***	***	*	***
D2	2022–2023	Control	4.69 b ± 0.08	20.3 b ± 0.46	19.5 b ± 1.05	0.64 b ± 0.02	4.91 b ± 0.05
		OM	5.03 a ± 0.08	23.9 a ± 0.65	24.6 a ± 0.67	0.70 a ± 0.02	5.37 a ± 0.04
		FF	4.74 b ± 0.08	20.1 b ± 0.65	19.9 b ± 0.65	0.65 b ± 0.02	4.95 b ± 0.08
		OM + FF	5.07 a ± 0.05	24.1 a ± 0.47	24.8 a ± 0.42	0.72 a ± 0.02	5.45 a ± 0.07
		*p*-value	***	***	***	***	***
	2023–2024	Control	4.81 b ± 0.07	20.6 b ± 0.53	20.6 b ± 0.46	0.62 b ± 0.02	4.98 b ± 0.06
		OM	5.12 a ± 0.05	24.9 a ± 0.35	25.8 a ± 0.61	0.71 a ± 0.03	5.47 a ± 0.13
		FF	4.87 b ± 0.05	20.7 b ± 0.60	20.8 b ± 0.32	0.62 b ± 0.03	4.94 b ± 0.07
		OM + FF	5.16 a ± 0.06	25.0 a ± 0.56	26.1 a ± 0.35	0.73 a ± 0.02	5.44 a ± 0.07
		*p*-value	**	***	***	**	***
D3	2022–2023	Control	5.00 b ± 0.05	20.3 b ± 0.57	24.4 b ± 0.60	0.58 b ± 0.03	5.21 b ± 0.11
		OM	5.19 a ± 0.03	24.7 a ± 0.80	27.8 a ± 0.31	0.68 a ± 0.03	5.62 a ± 0.03
		FF	5.00 b ± 0.06	20.6 b ± 0.36	24.7 b ± 0.36	0.59 b ± 0.01	5.23 b ± 0.07
		OM + FF	5.18 a ± 0.05	25.2 a ± 0.78	28.6 a ± 0.40	0.68 a ± 0.03	5.63 a ± 0.05
		*p*-value	**	***	***	**	***
	2023–2024	Control	4.97 b ± 0.04	19.5 b ± 0.56	23.9 b ± 0.64	0.59 b ± 0.03	5.30 b ± 0.09
		OM	5.15 a ± 0.03	24.3 a ± 0.40	27.2 a ± 0.30	0.68 a ± 0.03	5.66 a ± 0.05
		FF	5.00 b ± 0.04	19.9 b ± 0.47	23.7 b ± 0.40	0.58 b ± 0.03	5.27 b ± 0.05
		OM + FF	5.17 a ± 0.03	24.7 a ± 0.60	27.5 a ± 0.85	0.71 a ± 0.02	5.60 a ± 0.13
		*p*-value	***	***	***	**	***

D1, D2, and D3 indicate three different durian orchards in the study. OM, organic manure; FF, foliar fertilization; SOC, soil organic carbon; AP, available phosphorus; Ex. K^+^, soil exchangeable K; Ex. Ca^2+^, soil exchangeable Ca. Different letters indicate significance at *p* < 0.05 (*), *p* < 0.01 (**), and *p* < 0.001 (***). Mean ± standard deviation (n = 3).

**Table 2:** Mean values for leaf K and Ca concentrations under different amendments.

Site	Treatment	2022–2023		2023–2024	
		K (g kg^−1^)	Ca (g kg^−1^)	K (g kg^−1^)	Ca (g kg^−1^)
D1	Control	11.2 c ± 0.30	15.9 c ± 0.35	11.9 c ± 0.35	16.1 c ± 0.56
	OM	13.9 b ± 035	17.8 b ± 0.95	14.1 b ± 0.46	18.4 b ± 0.42
	FF	13.6 b ± 0.21	18.4 b ± 0.62	14.6 b ± 0.49	18.9 b ± 0.25
	OM + FF	15.8 a ± 0.36	20.5 a ± 0.87	16.9 a ± 0.67	20.4 a ± 0.70
	*p*-value	***	***	***	***
D2	Control	12.2 c ± 0.30	15.5 c ± 0.60	12.1 c ± 0.57	14.9 c ± 0.35
	OM	15.5 b ± 0.31	18.0 b ± 0.44	14.8 b ± 0.42	17.2 b ± 0.50
	FF	15.8 b ± 0.31	18.2 b ± 0.80	15.0 b ± 0.21	17.4 b ± 0.61
	OM + FF	16.9 a ± 0.75	20.9 a ± 0.36	17.9 a ± 0.20	19.9 a ± 0.35
	*p*-value	***	***	***	***
D3	Control	11.5 c ± 0.55	14.9 c ± 0.30	11.6 c ± 0.56	14.2 c ± 0.31
	OM	15.0 b ± 0.62	16.7 b ± 0.46	15.2 b ± 0.30	16.1 b ± 0.46
	FF	15.4 b ± 0.60	16.3 b ± 0.55	15.6 b ± 0.61	16.1 b ± 0.20
	OM + FF	17.6 a ± 0.40	19.7 a ± 0.47	17.8 a ± 0.42	19.9 a ± 0.67
	*p*-value	***	***	***	***

D1, D2, and D3 indicate three different durian orchards in the study. OM, organic manure; FF, foliar fertilization. Different letters indicate significance at *p* < 0.001 (***). Mean ± standard deviation (n = 3).

**Table 3:** Mean values for fruit proportions (peel, seeds, and arils) after different amendments.

Site	Treatment	2022–2023			2023–2024		
		Peel Proportion (%)	Seed Proportion (%)	Aril Proportion (%)	Peel Proportion (%)	Seed Proportion (%)	Aril Proportion (%)
D1	Control	68.1 a ± 0.35	8.80 ± 0.69	23.1 c ± 0.45	68.1 a ± 0.40	8.67 ± 1.07	23.2 c ± 0.72
	OM	67.8 a ± 0.46	7.80 ± 0.79	24.4 b ± 0.92	67.3 a ± 0.45	7.70 ± 0.36	25.0 b ± 0.47
	FF	68.2 a ± 0.50	7.30 ± 1.15	24.5 b ± 0.72	67.4 a ± 0.50	7.77 ± 0.71	24.9 b ± 0.49
	OM + FF	64.0 b ± 0.67	8.10 ± 0.80	27.9 a ± 0.21	64.4 b ± 0.67	7.30 ± 0.89	28.3 a ± 0.45
	*p*-value	**	ns	***	***	ns	***
D2	Control	68.4 a ± 0.70	7.93 ± 0.31	23.6 c ± 0.42	68.5 a ± 0.75	7.73 ± 0.84	23.8 c ± 0.38
	OM	66.9 a ± 1.00	7.47 ± 0.76	25.7 b ± 0.38	67.1 a ± 0.89	6.67 ± 1.16	26.2 b ± 0.31
	FF	67.2 a ± 1.51	7.10 ± 2.02	25.7 b ± 0.51	67.1 a ± 1.06	6.67 ± 0.70	26.2 b ± 0.42
	OM + FF	64.2 b ± 0.31	7.43 ± 0.40	28.4 a ± 0.26	64.2 b ± 0.42	7.50 ± 0.85	28.3 a ± 0.45
	*p*-value	**	ns	***	***	ns	***
D3	Control	67.7 a ± 0.80	8.50 ± 0.79	23.8 c ± 0.26	67.6 a ± 0.79	8.70 ± 0.56	23.7 c ± 0.40
	OM	66.7 a ± 0.57	7.13 ± 0.57	26.2 b ± 0.30	65.9 ab ± 1.18	8.43 ± 1.80	25.7 b ± 0.68
	FF	66.7 a ± 1.27	7.07 ± 1.24	26.3 b ± 0.40	65.4 b ± 1.08	8.73 ± 1.02	25.9 b ± 0.45
	OM + FF	63.0 b ± 0.50	8.43 ± 0.47	28.6 a ± 0.35	63.6 c ± 1.56	8.23 ± 1.46	28.2 a ± 0.36
	*p*-value	***	ns	***	**	ns	***

D1, D2, and D3 indicate three different durian orchards in the study. OM, organic manure; FF, foliar fertilization. Different letters indicate significance at *p* < 0.01 (**) and *p* < 0.001 (***); ns, not significant. Mean ± standard deviation (n = 3).

**Table 4:** Mean values for fruit yield and quality under different amendments.

Site	Season	Treatment	Fruit Yield (kg tree^−1^)	b* Value	TSS (%)	PD Rate (%)
D1	2022–2023	Control	65.1 c ± 1.35	38.8 c ± 2.03	27.3 c ± 0.56	12.6 a ± 0.81
		OM	70.6 b ± 0.90	45.0 b ± 1.46	29.2 b ± 0.30	8.37 b ± 0.42
		FF	71.9 b ± 2.31	47.2 b ± 1.05	29.2 b ± 0.31	8.53 b ± 0.55
		OM + FF	75.8 a ± 1.07	63.2 a ± 1.05	31.3 a ± 0.80	1.83 c ± 0.21
		*p*-value	***	***	***	***
	2023–2024	Control	90.1 c ± 1.39	42.3 c ± 1.43	26.9 c ± 0.40	12.9 a ± 1.35
		OM	94.2 b ± 0.47	48.4 b ± 0.75	29.0 b ± 0.46	8.23 b ± 0.31
		FF	94.5 b ± 0.55	49.5 b ± 0.65	29.2 b ± 0.40	8.77 b ± 0.31
		OM + FF	97.8 a ± 0.25	59.8 a ± 1.27	30.6 a ± 0.40	1.93 c ± 0.23
		*p*-value	***	***	***	**
D2	2022–2023	Control	67.3 c ± 0.35	42.4 c ± 0.82	27.4 c ± 0.56	11.7 a ± 0.86
		OM	70.8 b ± 0.46	49.2 b ± 2.17	29.1 b ± 0.26	8.40 b ± 0.70
		FF	70.3 b ± 1.69	49.2 b ± 1.23	29.3 b ± 0.67	8.63 b ± 0.70
		OM + FF	76.1 a ± 1.54	63.1 a ± 0.85	30.9 a ± 0.35	1.17 c ± 0.83
		*p*-value	***	***	***	***
	2023–2024	Control	89.5 c ± 1.40	37.3 c ± 11.8	27.3 c ± 0.32	13.4 a ± 1.20
		OM	94.9 b ± 0.31	50.8 b ± 1.25	28.8 b ± 0.36	8.60 b ± 0.90
		FF	95.3 b ± 0.96	50.7 b ± 1.22	28.9 b ± 0.47	8.43 b ± 0.61
		OM + FF	98.1 a ± 0.91	63.4 a ± 0.95	30.8 a ± 0.25	1.77 c ± 0.21
		*p*-value	***	**	***	***
D3	2022–2023	Control	66.8 c ± 1.72	42.1 c ± 1.73	26.9 c ± 0.47	14.2 a ± 0.66
		OM	71.0 b ± 1.42	47.7 b ± 1.16	28.9 b ± 0.30	8.87 b ± 0.35
		FF	71.7 b ± 1.65	50.2 b ± 1.83	28.8 b ± 0.65	8.57 b ± 0.81
		OM + FF	75.8 a ± 1.24	59.0 a ± 1.31	30.6 a ± 0.55	2.20 c ± 0.40
		*p*-value	***	***	**	**
	2023–2024	Control	88.3 c ± 1.56	43.4 c ± 0.95	27.8 c ± 0.36	14.4 a ± 0.97
		OM	93.3 b ± 0.97	52.2 b ± 1.15	29.8 b ± 0.31	9.53 b ± 0.72
		FF	94.0 b ± 0.79	52.9 b ± 1.65	30.2 b ± 0.61	9.53 b ± 0.65
		OM + FF	97.4 a ± 1.43	63.9 a ± 1.40	31.9 a ± 0.40	2.23 c ± 0.21
		*p*-value	***	***	**	***

D1, D2, and D3 indicate three different durian orchards in the study. TSS, total soluble solids; PD, physiological disorders; OM, organic manure; FF, foliar fertilization; b* (+yellowness, −blueness). Different letters indicate significance at *p* < 0.01 (**) and *p* < 0.001 (***). Mean ± standard deviation (n = 3).

**Table 5:** Pearson’s correlation coefficients (r values, n = 72) between soil and plant parameters.

**Soil pH**	**Soil pH**								
**SOC**	0.74 **	**SOC**							
**AP**	0.87 **	0.77 **	**AP**						
**Ex. K^+^**	0.51 *	0.72 **	0.42	**Ex. K^+^**					
**Ex. Ca^2+^**	0.77 **	0.76 **	0.79 **	0.75 **	**Ex. Ca^2+^**				
**Leaf K**	0.55 *	0.66 *	0.51 *	0.63 **	0.58 *	**Leaf K**			
**Leaf Ca**	0.38	0.60 *	0.39	0.38	0.25	0.77 **	**Leaf Ca**		
**Fruit yield**	0.37	0.17	0.16	0.18	0.17	0.31	0.17	**Fruit yield**	
**PD rate**	−0.54 *	−0.71 **	−0.52 *	−0.54 *	−0.46 *	−0.88 **	−0.91 **	−0.22	**PD rate**

SOC, soil organic carbon; AP, available phosphorus; Ex., exchangeable; PD, physiological disorders. * and ** indicate *p* < 0.05 and *p* < 0.01, respectively.

## Data Availability

Data are contained within the article.

## Supplementary Materials

The following supporting information can be downloaded at https://www.mdpi.com/article/10.3390/plants14081185/s1: Table S1: Initial soil physicochemical traits of study sites; Table S2: Color characteristics of durian aril under different amendments.

## Abbreviations

The following abbreviations are used in this manuscript:

OM	Organic manure
FF	Foliar fertilization
PD	Physiological disorders
SOC	Soil organic carbon
TSS	Total soluble solids
AP	Available phosphorus
VMD	Mekong Delta, Vietnam
