# Supplementary material for: Combining Organic and Foliar Fertilization to Enhance Soil Fertility and Mitigate Physiological Disorders of Durian (Durio zibethinus Murr.) Fruit in the Tropics
Source: Plants (Basel). 2025 Apr 11;14(8):1185. doi: 10.3390/plants14081185 (PMC12030457; doi:10.3390/plants14081185)
Supplement: Supplementary file 1 [file plants-14-01185-s001.zip › plants-3546595-Supplementary file.pdf]

Supplementary file

Combining Organic and Foliar Fertilization to Enhance Soil Fertility and Mitigate Physiological Disorders of Durian (*Durio zibethinus* Murr.) Fruit in the Tropics

Le Van Dang [1,2], Nguyen Kim Quyen [3,*], Ngo Phuong Ngoc [1], Le Minh Ly [1], Pham Thi Phuong Thao [1], and Ngo Ngoc Hung [1]

[1] College of Agriculture, Can Tho University, Can Tho City 94000, Vietnam
[2] United Graduate School of Agricultural Science, Tokyo University of Agriculture and Technology, Tokyo 183-8509, Japan
[3] Faculty of Agriculture and Fishery, University of Cuu Long, Vinh Long 85000, Vietnam

* Correspondence: nguyenkimquyen@mku.edu.vn (N.K.Q.); Tel.: +84-918779836

Table S1. Initial soil physicochemical traits of study sites.

Property	Unit	Study location		
		**D1**	**D2**	**D3**
pH_{H2O} (1:2.5)	-	4.82	4.77	4.94
EC (1:2.5)	mS cm^{-1}	0.12	0.18	0.16
Avail.P	mgP kg^{-1}	25.9	20.5	24.6
SOC	g kg^{-1}	19.5	18.8	17.3
Exchangeable cations				
Na^{+}	$cmol_c$ $100g^{-1}$	0.35	0.40	0.44
K^{+}	$cmol_c$ $100g^{-1}$	0.52	0.66	0.59
Ca^{2+}	$cmol_c$ $100g^{-1}$	4.85	5.02	5.33
Mg^{2+}	$cmol_c$ $100g^{-1}$	5.62	5.09	5.77
CEC	$cmol_c$ $100g^{-1}$	18.3	17.8	18.5
Soil BD	g cm^{-3}	1.06	1.11	1.05
Soil particles				
Sand	%	0.8	1.3	1.2
Silt	%	44.3	45.5	44.4
Clay	%	54.9	53.2	54.4

D1, D2, and D3 indicate three different durian orchards in the study. EC: electrical conductivity, Avail.P: available phosphorus, Tot.N: total nitrogen, SOC: soil organic carbon, CEC: cation exchange capacity, BD: bulk density.

Table S2. Color characteristics of durian aril under different amendments.

Site	Treatment	2022–2023		2023–2024	
		L*	a*	L*	a*
D1	Control	82.1 ± 1.39	12.3 ± 0.82	78.6 ± 2.57	13.5 ± 1.23
	OM	80.6 ± 0.65	12.4 ± 1.41	79.7 ± 3.02	13.7 ± 1.11
	FF	81.8 ± 3.12	12.9 ± 1.78	79.8 ± 4.81	13.6 ± 0.70
	OM + FF	81.9 ± 1.79	14.0 ± 1.51	79.6 ± 6.11	13.5 ± 0.67
	p-value	ns	ns	ns	ns
D2	Control	81.2 ± 3.01	13.3 ± 1.07	82.3 ± 4.05	14.3 ± 0.82
	OM	79.9 ± 3.74	13.3 ± 2.35	83.6 ± 3.43	13.5 ± 1.15
	FF	81.2 ± 4.21	12.9 ± 1.57	82.4 ± 3.80	13.5 ± 0.87
	OM + FF	81.7 ± 3.44	13.1 ± 2.15	82.5 ± 2.57	13.2 ± 1.73
	p-value	ns	ns	ns	ns
D3	Control	80.5 ± 3.67	12.4 ± 0.91	79.8 ± 3.41	13.6 ± 0.95
	OM	80.1 ± 3.08	12.6 ± 0.95	80.8 ± 2.72	13.9 ± 0.26
	FF	81.7 ± 4.38	12.4 ± 1.10	79.7 ± 3.45	13.2 ± 1.42
	OM + FF	81.0 ± 2.27	12.9 ± 0.49	79.2 ± 3.45	13.5 ± 1.19
	p-value	ns	ns	ns	ns

D1, D2, and D3 indicate three different durian orchards in the study. TSS, total soluble solids; PD, physiological disorders; OM, organic manure; FF, foliar fertilization; L* (lightness intensity); a* (+ redness, − greenness); ns, not significant. Mean ± standard deviation (n = 3).
